# Isolation and characterization of methicillin-resistant *Staphylococcus aureus* phage SPB against MRSA planktonic cells and biofilm

**DOI:** 10.3389/fmicb.2025.1554182

**Published:** 2025-05-21

**Authors:** Lan Ma, Yingyu Liu, Xiaofeng Zheng, Baili Zheng, Yaling Cheng, Yuxuan Cai, Yongchao Li, Wei Zhang

**Affiliations:** ^1^College of Veterinary Medicine, Xinjiang Agricultural University, Ürümqi, China; ^2^Xinjiang Key Laboratory of New Drug Research and Development for Herbivores, Ürümqi, China; ^3^College of Veterinary Medicine, Nanjing Agricultural University, Nanjing, China

**Keywords:** Methicillin-resistant *Staphylococcus aureus*, *Staphylococcus aureus* phage, virulent phage, biofilm, antibacterial agent

## Abstract

Methicillin-resistant *Staphylococcus aureus* (MRSA) is a common antibiotic-resistant pathogen. MRSA and its biofilm pose a great threat to the food industry. In this study, we characterized the biological properties and antibacterial efficacy of phages through the double-layer plate method, transmission electron microscopy (TEM), whole-genome sequencing (WGS), bioinformatic analyses, fluorescence microscopy, and biofilm eradication assays. The results demonstrated that phage SPB is a virulent member of the genus *Kayvirus* (subfamily *Twortvirinae*), exhibited a broad host range spanning *Staphylococcus* species. It effectively lysed 97.3% (36/37) of clinical MRSA isolates and 100.0% (10/10) of coagulase-negative *staphylococci* strains tested. The optimal multiplicity of infection (MOI) was determined to be 1, with a latent period of 10 min. Environmental stability assays revealed that phage SPB maintained infectivity across temperatures ranging from 4°C to 50°C and pH values between 4 and 11. Genomic analysis showed that phage SPB possesses a 143,170 bp genome with a G+C content of 30.2%, encoding 218 putative coding sequences (CDSs), 3 tRNAs, and no virulence factors were identified through in software screening. Phage SPB exhibited potent inhibition of planktonic bacterial growth. Furthermore, at varying multiplicities of infection (MOIs), it significantly suppressed biofilm formation and eradicated pre-existing biofilms, with statistical significance (*P* < 0.001). These results suggest that phage SPB can be used as a potential antimicrobial agent to prevent and remove MRSA and its biofilm from food processing.

## 1 Introduction

Methicillin-resistant *Staphylococcus aureus* (MRSA) is resistant to Methicillin with encoded the *mecA* gene the *Staphylococcus aureus* (*S. aureus*) (Graveland et al., 2011). Initially, MRSA was mainly prevalent in hospitals (HA-MRSA), but in the late 1990s, MRSA was associated with community infections (CA-MRSA) (Stefani et al., 2012). Currently, MRSA has been found to be associated with livestock infection (LA-MRSA) (Lakhundi and Zhang, 2018). Studies have demonstrated that LA-MRSA is not only present in animals but can also be transmitted to humans, particularly affecting occupationally exposed workers in the livestock (Cuny et al., 2009). Furthermore, the presence of MRSA has been documated in various livestock (Devriese et al., 1972; Nemati et al., 2008; Idelevich et al., 2016; Jauro et al., 2022), poultry, animal food (Abolghait et al., 2020), and food processing environments (Sadiq et al., 2020). MRSA is not only high prevalent but also capable of causing severe infections associated with high mortality. Clinically, MRSA can cause a range of organ-specific infections, with the skin and subcutaneous tissue being the most commonly affected, followed by more aggressive infections such as osteomyelitis, meningitis, pneumonia, lung abscess, and empyema (Turner et al., 2019; Nandhini et al., 2022). The U.S. Centers for Disease Control and Prevention (CDC) has reported that MRSA poses a serious threat to human health, with escalating concerns regarding drug resistance, treatability, mortality, prevention, and transmissibility (Nandhini et al., 2022). Consequently, there is an urgent need to develop new antimicrobial alternatives to combat drug-resistant MRSA infections.

Bacterial biofilm (BF) refers to an organized group of bacteria that are attached to the surfaces of living or inanimate objects and are surrounded by extracellular macromolecules (Jiang et al., 2021). Compared to floating bacteria, BF bacteria exhibit significantly enhanced environmental tolerance, with antibiotic resistance escalating over 1000-fold (Saginur et al., 2006). General antibacterial agents are difficult to penetrate the extracellular lipopolysaccharide matrix layer of BFs, which makes it difficult to eliminate BFs in the livestock, food processing, and in clinical settings through conventional methods (Yang et al., 2018).

Bacteriophages, first discovered in the early 20th century, are the most abundant organisms in the world (Yang et al., 2018). Phage therapy offers several advantages, including high bacterial specificity (Shkoporov et al., 2019), reduced bacterial tolerance, and biofilm eradication capacity (Gutierrez et al., 2015; Song et al., 2021). These phages exclusively infect bacteria and are non-pathogenic to humans, rendering them safe for clinical and food applications (Gutierrez et al., 2016). For instance, the *S. aureus* phage mixture AB-SA01 demonstrated effectiveness in treating wound infections caused by MRSA in diabetic mice (Kifelew et al., 2020). *S. aureus* phage mixtures can be safely administered to patients with *S. aureus* infections (Petrovic Fabijan et al., 2020). Furthermore, phages are capable of targeting and disrupting biofilms. For example, phages LAh1-LAh10 significantly reduced the number of Aeromonas hydrophila biofilms after 24 h of treatment (Kabwe et al., 2020), while *S. aureus* phage JS02 reduced biofilm formation by ~68% (Zhang et al., 2022). Although the high specificity of phages presents unique advantages, their narrow host range and the need for high personalization, pose significant barriers to the utilization of phage-based applications (Lu et al., 2023). Consequently, there is an urgent need to screen for highly efficient phages that possess a broad host range. Such phages are not only ideal candidates for phage therapy but also enhance the selection of available phage resources.

In this study, MRSA phages were isolated, and one phage, designated SPB, which exhibited the broadest host range, was selected for comprehensive analysis of its biological properties and genome sequence. The therapeutic potential of the phage SPB was evaluated by determining its inhibitory effect on planktonic bacteria as well as its effect on the biofilm. In this study, we aim to screen a broad-spectrum potent phage with a view to becoming a candidate for antibiotic use and enriching the phage library.

## 2 Materials and methods

### 2.1 Samples and bacterial strains

The strains used in this study are detailed in Supplementary Table S1. All strains were cultured in a 7.5% Sodium chloride broth at 37°C and subsequently stored at −80°C in a 1:1 mixture with 60% glycerol. Phage samples were obtained from the effluent of a pigeon farm located in Xinjiang.

### 2.2 Isolation and purification of phage

Phage isolation and purification were conducted using the double-layer plate method, as described in previous studies (Kong et al., 2022). Sewage samples were initially centrifuged at 5,000 rpm for 10 min and then filtered through a 0.22 μm filter (Merck Millipore, Germany) to eliminate bacterial contaminants. To isolate phages, 1 mL of B2-2 culture (2 × 10^7^ CFU/mL) was combined with 10 mL of fresh LB broth and 1 mL of the filtered sewage sample. The mixture was incubated overnight at 37°C with shaking at 180 rpm. Following incubation, the mixture was centrifuged at 5,000 rpm for 10 min, and the supernatant was filtered through 0.22 μm pore size filters. Next, 100 μL filtrate was mixed with 100 μL of log-phase host bacteria and allowed to incubate at room temperature for 10 min. The resulting mixture was then added to 7 mL LB medium containing 0.6% agar, poured onto LB solid agar plates, and incubated overnight at 37°C. Phage plaques were observed and a single plaque was picked for further purification, which was repeated 3–5 times using the double-layer plate method.

### 2.3 Determination of phage host range

Determination of phage host range was performed by reference (Li et al., 2020). Information on the strains used in the experiment is listed in the Supplementary Table S1. The test strains were cultured to the logarithmic phase of growth (OD_600_ = 0.2–0.3). 100 μL aliquot of early log-phase bacterial culture was mixed with 7 mL LB medium containing 0.7% agar and overlaid onto LB plates (1.5% agar), following, 7 μL droplets of phage lysate (~10^8^ PFU/mL) were spotted onto each plate. Following overnight incubation at 37°C, plaque formation was assessed visually. The phages host range profiles were visualized using the ChiPlot online website (https://www.chiplot.online/tvbot.html).

### 2.4 Phage transmission electron microscopy (TEM)

The purified phage particles with a concentration of × 10^8^ PFU/mL were dropped onto a glass slide 200 mesh copper film. Subsequently, they were negatively stained with 2% (w/v) phosphotungstate (PTA). After allowing the samples to dry naturally for 20–30 min, the phage particles were observed using a HT7700 (HITACHI, Japan) TEM at an accelerated voltage of 80 kV.

### 2.5 Determination of the optimal multiplicity of infection (MOI) of the phage

The optimal MOI is a parameter frequently employed in studies of virus-infected cells and refers to the ratio of virus particles to host cells at the time of infection (Jiang et al., 2021). In brief, 100 μL of bacterial culture was mixed with 100 μL of phage at various concentration ratios (10, 1, 0.1, 0.01, 0.001, and 0.0001). The mixture was allowed to stand at room temperature for 10 min, followed by incubation in a shaker at 37°C, 180 r/min for 4 h. Phage potency was determined by the double-layer plate method, and the MOI that produced the highest phage titer was determined to be the optimal MOI. The experiment was repeated three times.

### 2.6 Determination of phage one-step growth curves

The one-step growth curve of the phage was plotted following the method described in the literature (Zhang et al., 2017). Briefly, the phage at the optimal MOI was co-cultured with bacteria for 10 min to ensure complete adsorption. Unadsorbed phages were removed by centrifugation, and the resulting pellet was resuspended in 4 mL of fresh LB medium. The suspension was incubated at 37°C with shaking at 180 rpm. The phage titer was measured every 10 min using the double-layer plate method. The experiment was repeated three times.

### 2.7 Temperature and pH stability of phages

Temperature stability was assessed according to the procedure outlined in the literature (Yang et al., 2022). To evaluate the temperature sensitivity of the phage, 500 μL of phage solution was incubated in a water bath at temperatures of 4, 30, 40, 50, 60, and 70°C. Samples were collected at 20, 40, and 60 min, and their phage titer was immediately determined using the double-layer plate method. The experiment was repeated 3 times. To evaluate the pH stability of the phage, 100 μL of phage was mixed with 900 μL of LB liquid medium at varying pH values (2, 3, 4, 5, 6, 7, 8, 9, 10, 11, 12, and 13) and incubated at 37°C for 1 h. Phage potency was then assessed using the double-layer plate method. The experiment was repeated three times.

### 2.8 Phage DNA extraction and genome sequencing

The phage DNA genome was extracted using the HiPure Viral DNA Mini Kit (D3191-02, Magen, China). Complete genome sequencing was performed on the Illumina HiSeq platform (Illumina, San Diego, CA, USA, United States). Raw sequencing data were processed and filtered using Trimmomatic to generate clean data. Genome assembly was then carried out using SPAdes.

### 2.9 Annotation of the genome

The entire genome of the phage was annotated using Prokka online software, and genome mapping was performed using Proksee 1.2.0^2^. Phage life cycle prediction, along with the identification of Anti-CRISPR and antimicrobial resistance genes, was conducted using PhageScope (McNair et al., 2012). Virulence factor distribution was analyzed using the VFDB database (Liu et al., 2019). To identify phages genetically related to SPB, a search was performed in the NCBI database using online BLASTn. Phage genome comparison results were visualized using EasyFig software (Krzywinski et al., 2009).

### 2.10 Phylogenetic analysis

The amino acid sequences of the conserved terminal enzyme large subunit (TerL) in phage were compared using BLASTp. On the basis of the comparison results, a Neighbor-joining (NJ) phylogenetic tree was constructed using the Poisson model with MEGA 6.0 software, and Bootstrap values were calculated based on 1,000 replicates. The phylogeny was beautified using the online software ChiPlot (https://www.chiplot.online/).

### 2.11 Lytic efficiency of phage SPB with different MOI on planktonic bacteria

The experiment was conducted with reference to the method described by Cui et al. (2017). The MRSA strains to be tested were cultured overnight in LB broth. On the following day, 30 ml of fresh LB broth was inoculated with 1% MRSA overnight culture. The MRSA strains was incubated at 37°C, 180 r/min until logarithmic phase of growth. SPB was added at an MOI of 100, 10, and 1 and incubated at 37°C with shaking at 180 r/min in an incubator. The OD_600_ values of the mixture were measured every 20 min, and a phage-free treatment was used as the control. The experiment was repeated three times.

### 2.12 Assessment of the effects of phage SPB on biofilm formation

The inhibitory effect of phage SPB on biofilm formation and its ability to remove biofilm were evaluated using previously reported methods with minor modifications. A 96-well cell culture plate was used for the experiment. In each well, add 100 μL of MRSA at a final concentration of 10^6^ CFU/mL. Phage SPB (100 μL) was then added to the MRSA culture at MOI of 100, 10, 1, 0.1, and 0.01. The cultures were incubated at 37°C for 24 h. For biofilm removal experiments, MRSA was first incubated at 37°C for 24 h to allow biofilm formation, and then treated with SPB at concentrations of 10^8^, 10^7^, 10^6^, 10^5^, and 10^4^ CFU/mL for 18 h. The positive control (untreated) received an equal volume of untreated SM buffer, while the negative control (control) was treated with 200 μL of TSB medium per well. Biofilm inhibition and removal by SPB were assessed using crystal violet staining. Each well was rinsed three times with 200 μL of sterile PBS and dried at 56°C for 1 h. Then, 200 μL of 1% crystal violet solution was added to each well and incubated for 30 min. The crystal violet solution was discarded, and the wells were washed 3 to 5 times with 200 μL of sterile PBS before being dried at 56°C for 1 h. Finally, 200 μL of anhydrous ethanol was added to dissolve the crystal violet, and the OD_600_ measured with a Optical Absorption Microplate Reader (Beijing Longyue Biotechnology Development Co., Ltd., Beijing, China). All the experiments were performed in triplicate.

### 2.13 Microscopic imaging of biofilms

Mature biofilms were cultured on 24-well cell culture plate. The medium was removed and the biofilm was washed three times with PBS to remove the planktonic bacteria on the surface, and then the biofilm was treated with 2 mL of phage with potencies of 10^4^, 10^5^, 10^6^, 10^7^, 10^8^ PFU/mL. In the control group, 2 mL of SM buffer was added and the biofilm samples were collected after 18 h of treatment. The treated biofilm was washed gently with PBS for 3 times and fixed with formaldehyde for 10 min, after which the excess formaldehyde was removed by washing with PBS. The samples were stained with DAPI for 20 min and finally observed under a fluorescence microscope.

### 2.14 Statistical analysis

IBM SPSS Statistics 27 software was used to complete the statistical analysis, and the one-way ANOVA test was used to compare the experimental group with the control group, *P* < 0.05 was considered statistically significant. The data were expressed as mean ± Standard deviation (SD) and images were plotted using the GraphPad Prism 8 software package.

## 3 Results

### 3.1 The host range of phage

**Figure 1:**
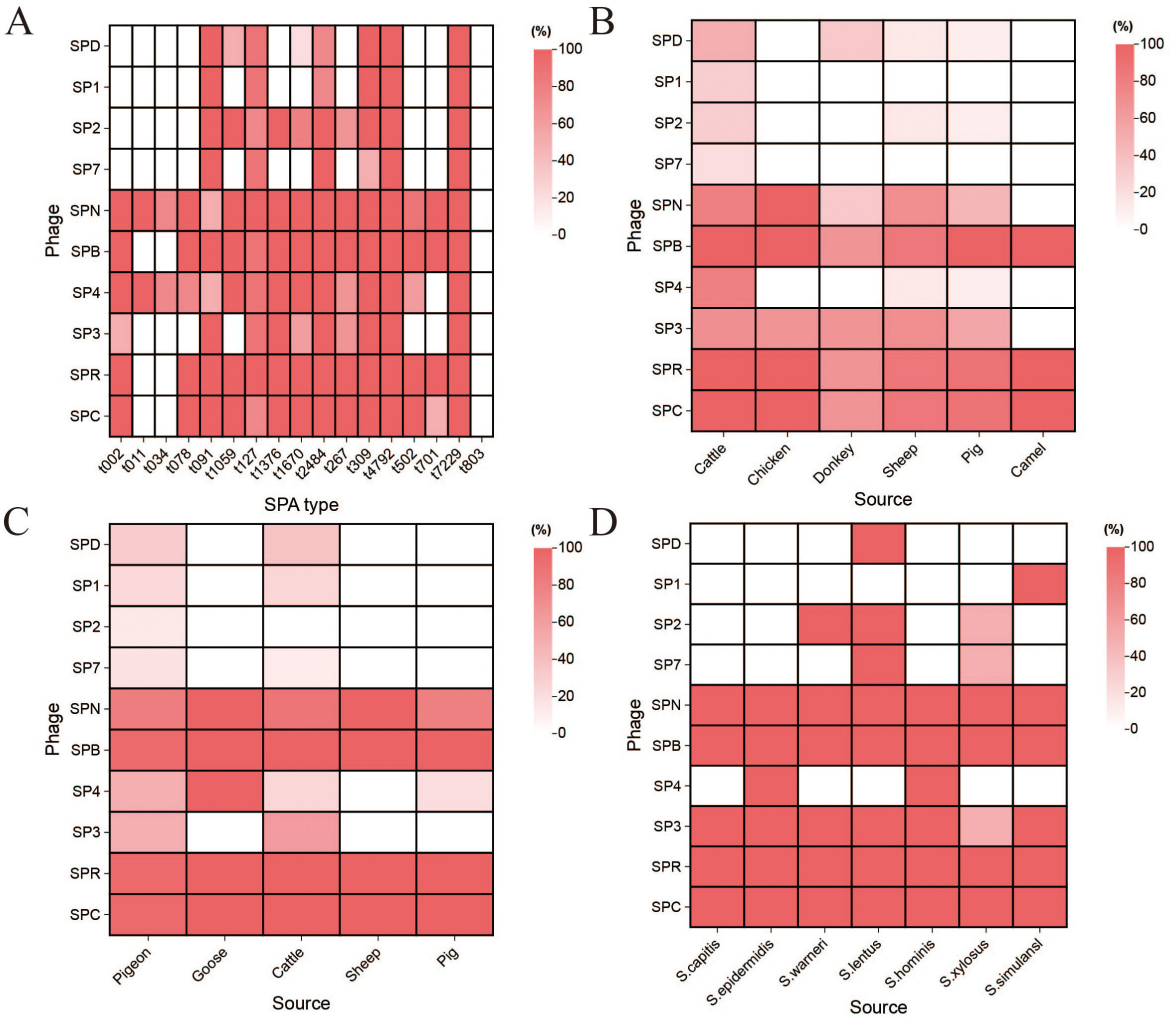
The host range of the phage. **(A)** Host range of the isolated phages to the *S. aureus* from different SPA type. **(B)** Host range of the isolated phages to the *S. aureus* from different sources. **(C)** Host range of the isolated phages to the MRSA isolates of different animal origins. **(D)** Host range of the isolated phages to the *staphylococci*.

From June to September 2023, 22 sewage samples were collected from the sewage treatment plant of the Urumqi Hualing Slaughterhouse and the new pigeon industry in Kashgar, Xinjiang. Using Pigeon-origin MRSA as the host bacterium, 10 phages were isolated from the sewage samples using the double agar method, namely SPD, SP1, SP2, SP7, SPN, SPB, SP4, SP3, SPR, and SPC (Figure 1 and Supplementary Table S1).

The host range of the ten phages was determined using the spot test to identify those with broad-spectrum and efficient lytic activity. The results of phage lysis against 60 *S. aureus* strains with 17 different SPA types (Figure 1A and Supplementary Table S1) showed that phage SPN could lyse 16 different SPA-types *S. aureus*, phage SP4 could lyse 15 SPA-types *S. aureus*, while phages SPB, SPR, and SPC could lyse 14 SPA-types each. Phages SP2, SP3, SPD, SP1, and SP7 demonstrated lytic activity against 10, 10, 8, 6, and 6 SPA-types *S. aureus*, respectively.

Further testing of phage lysis against 33 *S. aureus* strains from different sources (Figure 1B and Supplementary Table S1) revealed that phage SPB could lyse *S. aureus* strains from cattle, chicken, pig, and camel, while SPR and SPC could lyse *S. aureus* strains from cattle, chicken, and camel. SPN was able to lyse *S. aureus* strains from chicken, while other phages exhibited varying degrees of lytic activity, though the effects were less pronounced.

The lytic activity of phages against 37 strains of MRSA from different sources (Figure 1C and Supplementary Table S1) demonstrated that phages SPB, SPR, and SPC could lyse 97.3% (36/37) of MRSA strains from various animal sources. Phages SPN, SPD, SP1, SP2, SP7, SP4, and SP3 were able to lyse 83.8% (31/37), 27.0% (10/37), 18.9% (7/37), 8.1% (3/37), 13.5% (5/37), 43.2% (16/37), and 45.9% (17/37) of the MRSA strains.

The range of phage lysis of 10 coagulase-negative *staphylococci* is shown in Figure 1D and Supplementary Table S1. Phages SPN, SPB, SPR and SPC all lysed 10 strains of *staphylococci*, and phage SP3 lysed 9 strains of *staphylococci*. Phages SPD and SP1 were effective against only one *staphylococci* species. Based on these results, phage SPB exhibited a wide host range, making it the primary focus of subsequent experiments.

### 3.2 Phage isolation and morphology

**Figure 2:**
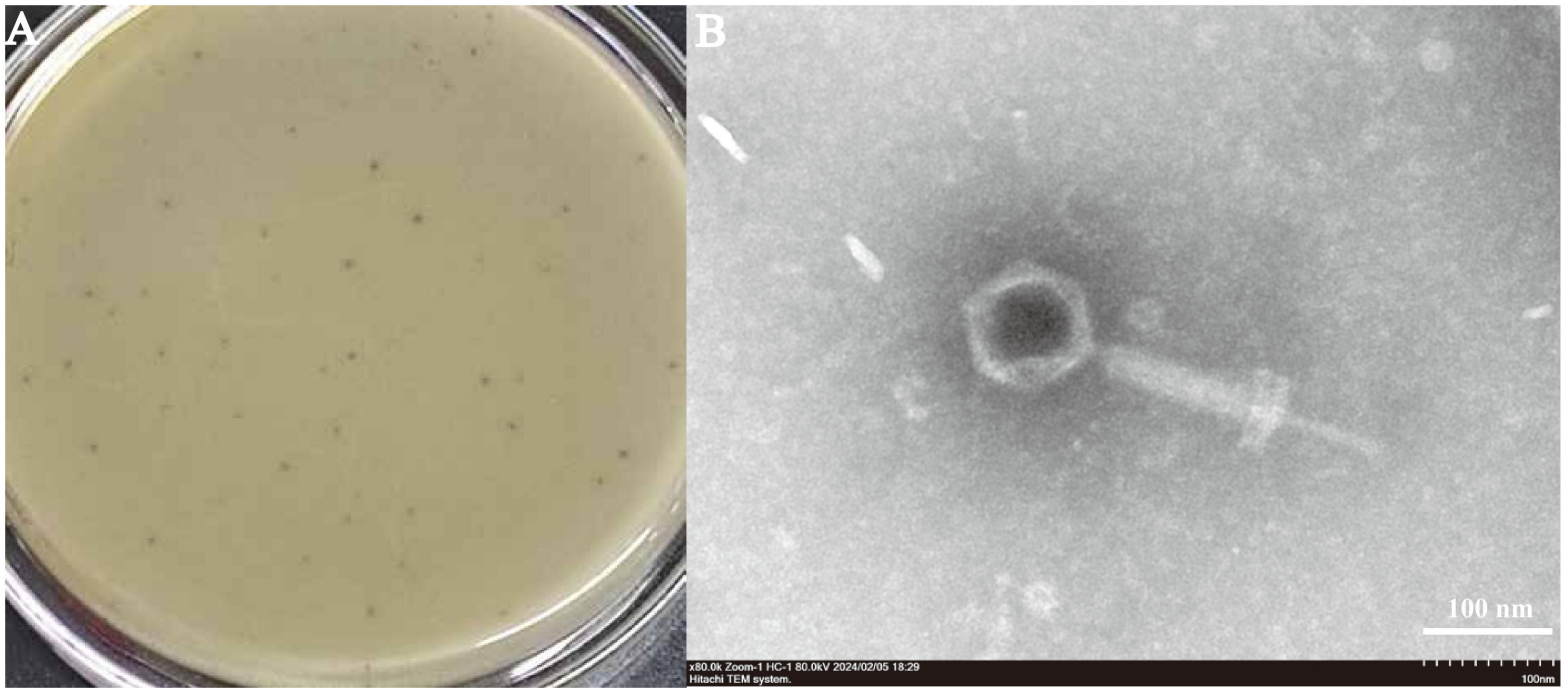
**(A)** Plaque morphology of phage SPB: Plaque morphology: Forms plaques 0.5–1 mm in diameter, which are hyaline and have neat edges. **(B)** Phage morphology by scanning transmission electron microscopy. SPB had an icosahedral capsid and a contractile tail.

To examine the morphology and structure of the phages. The phage SPB plaques, which ranged from 0.5 to 1 mm in diameter, exhibited translucent centers and well-defined edges (Figure 2A). After propagation, the phage titer reached 1.61 × 10^8^ PFU/mL. TEM analysis revealed that SPB had an icosahedral capsid with a diameter of 84 ± 1 nm and a contractile tail measuring 210 ± 1 nm (Figure 2B). Based on these morphological characteristics, the phage was classified as belonging to the family *Myoviridae*.

### 3.3 The optimal MOI and one-step growth curve

**Figure 3:**
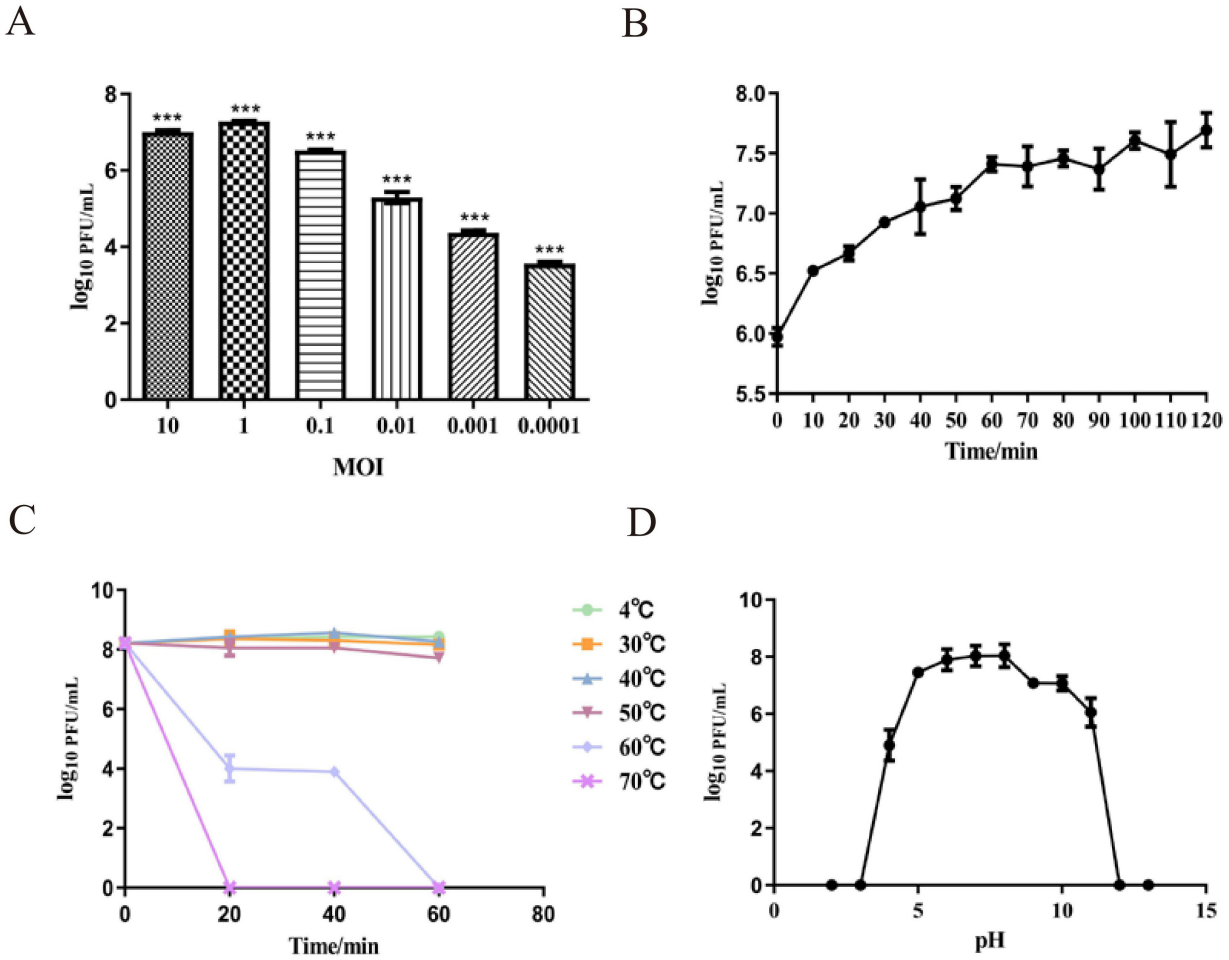
Growth characteristics: the optimal MOI and one-step experiments. **(A)** The Optimal Multiplicity of Infection (MOI) phage SPB was tested under different MOIs (10, 1, 0.1, 0.01, 0.001, 0.0001). When at an MOI of 1, phage SPB produced highest phage titer. ***P < 0.001. **(B)** The one-step growth curve of phage SPB. Sensitivity of Temperature and PH. **(C)** Sensitivity of temperature: phage SPB suspension was incubated in different temperatures (4, 30, 40, 50, 60, 70°C) for 20 30 min or 1 h. **(D)** Sensitivity of PH: phage SPB suspension was incubated in different PH conditions for 1 h.

To determine the optimal MOI between phage SPB and its host bacterium. As shown in Figure 3A, the highest phage titer of ~1.9 × 10^7^ PFU/mL was observed at an MOI of 1. Therefore, an MOI of 1 was determined to be the optimal infection ratio for phage SPB. Result display (Figure 3B) that the latent period of phage SPB was about 10 min, and the cleavage period was 60 min, and the amount of cleavage was about 5.46 PFU/CFU.

### 3.4 Thermo and pH Stability

To identify the stability profile of phage SPB. Thermal stability test results (Figure 3C) showed that phage SPB maintained relatively stable activity within a temperature range of 4°C to 50°C. At 60°C, the survival rate declined, with complete inactivation occurring after 1 h. At 70°C, the phage was fully inactivated within 20 min. The phage was stored in a refrigerator at 4°C, and its potency was determined to be 2.8 × 10^6^ PFU/mL after 2 months.

The pH stability results showed in Figure 3D. The highest survival rate was observed at pH value 6–8, indicating that this pH range is optimal for phage SPB. The phage's survival rate reduced as acidity and alkalinity increased, and phage SPB was completely inactivated under acidic (pH < 4) or highly alkaline (pH > 12) conditions.

### 3.5 Basic genomic features of SPB

**Figure 4:**
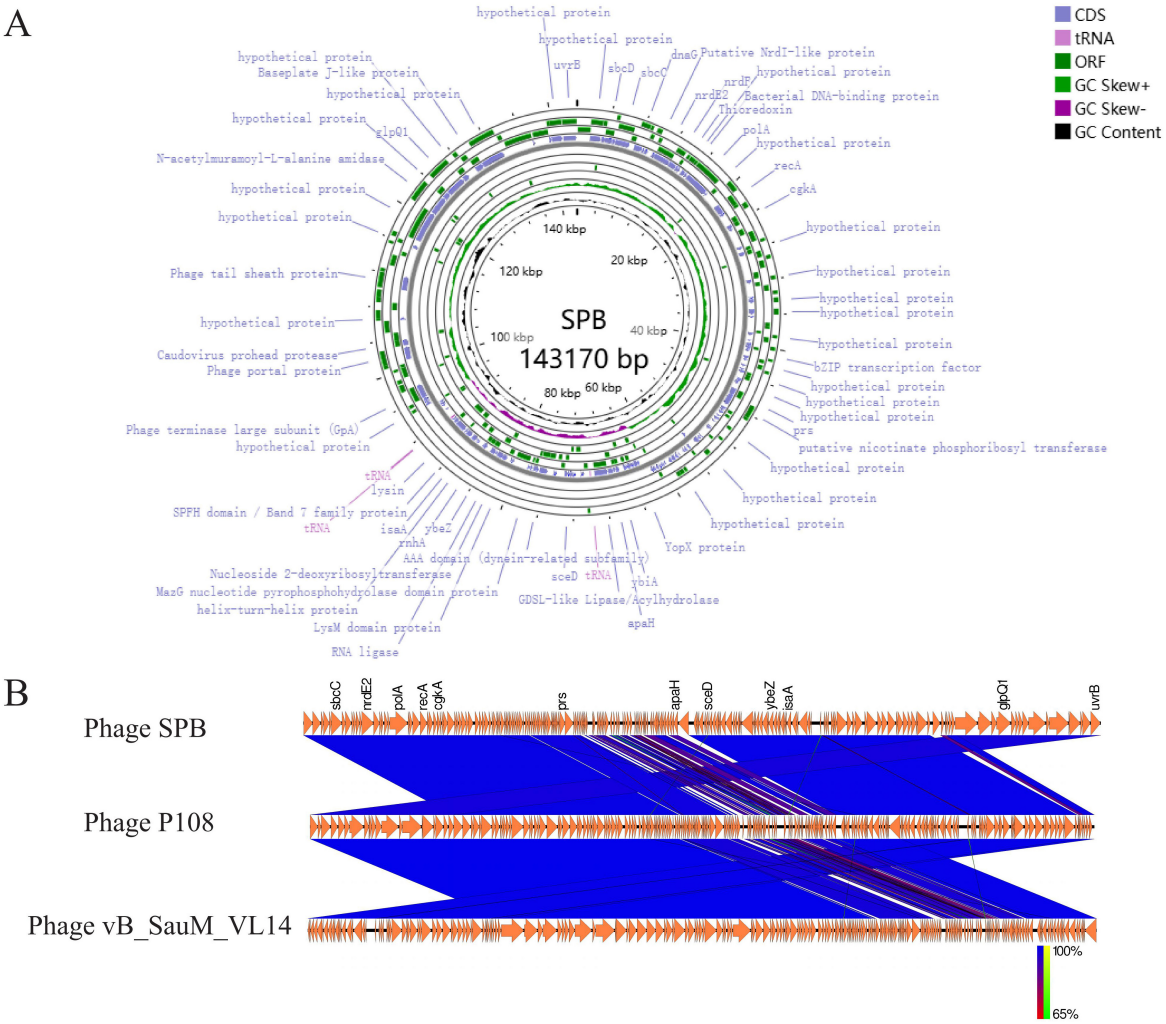
Genome analysis. **(A)** Phage SPB genome map. **(B)** After obtaining CDS annotations through Prokka, a collinearity analysis graph was drawn. Comparison of phage SPB with the highly homologous phages P108 and vB_SauM_VL14.

In order to understand the genetic characteristics of phage SPB in detail, we sequenced and analyzed the phage for genomic characterization and safety. The accession number for SPB in the NCBI database is PQ777470. Phage SPB is a double-stranded DNA phage (dsDNA) with a complete genome of 143,170 bp and a GC content of 30.2%. The total length of the genes encoded by phage SPB is 128,603 bp, with an average gene length of 589.92 bp. The lengths of individual genes range from 72 bp to 4,056 bp. The complete genome structure of phage SPB is illustrated in Figure 4A. A total of 218 coding sequences (CDS) were predicted using Prokka, of which 157 were located on the positive strand and 61 on the negative strand. Among these, 43 CDSs were homologous to genes encoding proteins with known functions, while the remaining sequences were annotated as encoding hypothetical proteins. Prokka also predicted three tRNAs for phage SPB, namely, methionine, aspartic acid, and phenylalanine. Additionally, an enzyme predicted to hydrolyze the bacterial cell wall peptidoglycan, identified as lyase, is thought to work in conjunction with holin protein to form a binary cleavage system for host cell lysis. The genome also contains a tail hydrolase, which is responsible for localized cell wall degradation following phage adsorption, facilitating the injection of nucleic acids into the host cell. Finally, three anti-CRISPR proteins were identified through PhageScope prediction, suggesting a potential mechanism for evading host immune responses.

The complete genome of phage SPB was compared with sequences in the NCBI database, revealing a high degree of homology with *S. aureus* phages of the genus *Kayvirus* in GenBank, with coverage and similarity exceeding 90%. The two phages with the highest homology were selected for a comparative genome analysis (Figure 4B). This comparison revealed significant differences in the local gene content of SPB compared to the other two phages, and the overall gene arrangement also varied, which made it a new strain of phage.

### 3.6 Phylogenetic analysis

**Figure 5:**
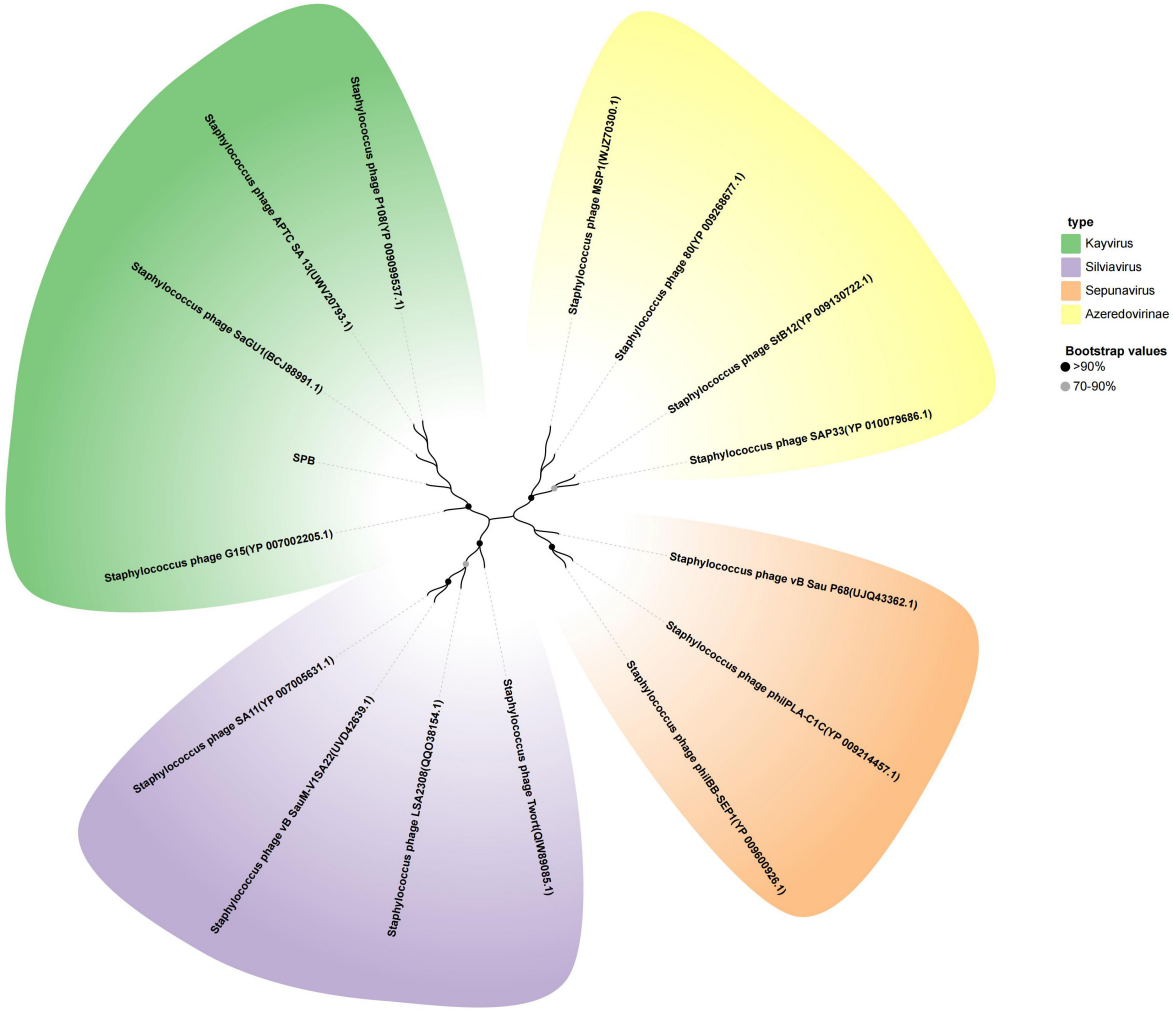
Neighbor-joining phylogenetic tree based on the terminase large subunit amino acid sequence.

To further analyze the phylogenetic position of SPB, the Phage terminase large subunit was analyzed using MEGA-X software to construct a phylogenetic tree (Figure 5). The results showed that SPB belongs to the genus *Kayvirus* together with phage P108 (YP 009099537.1) and phage GH15 (YP 007002205.1).

### 3.7 Lytic efficiency of phage SPB with different MOI on planktonic bacteria

**Figure 6:**
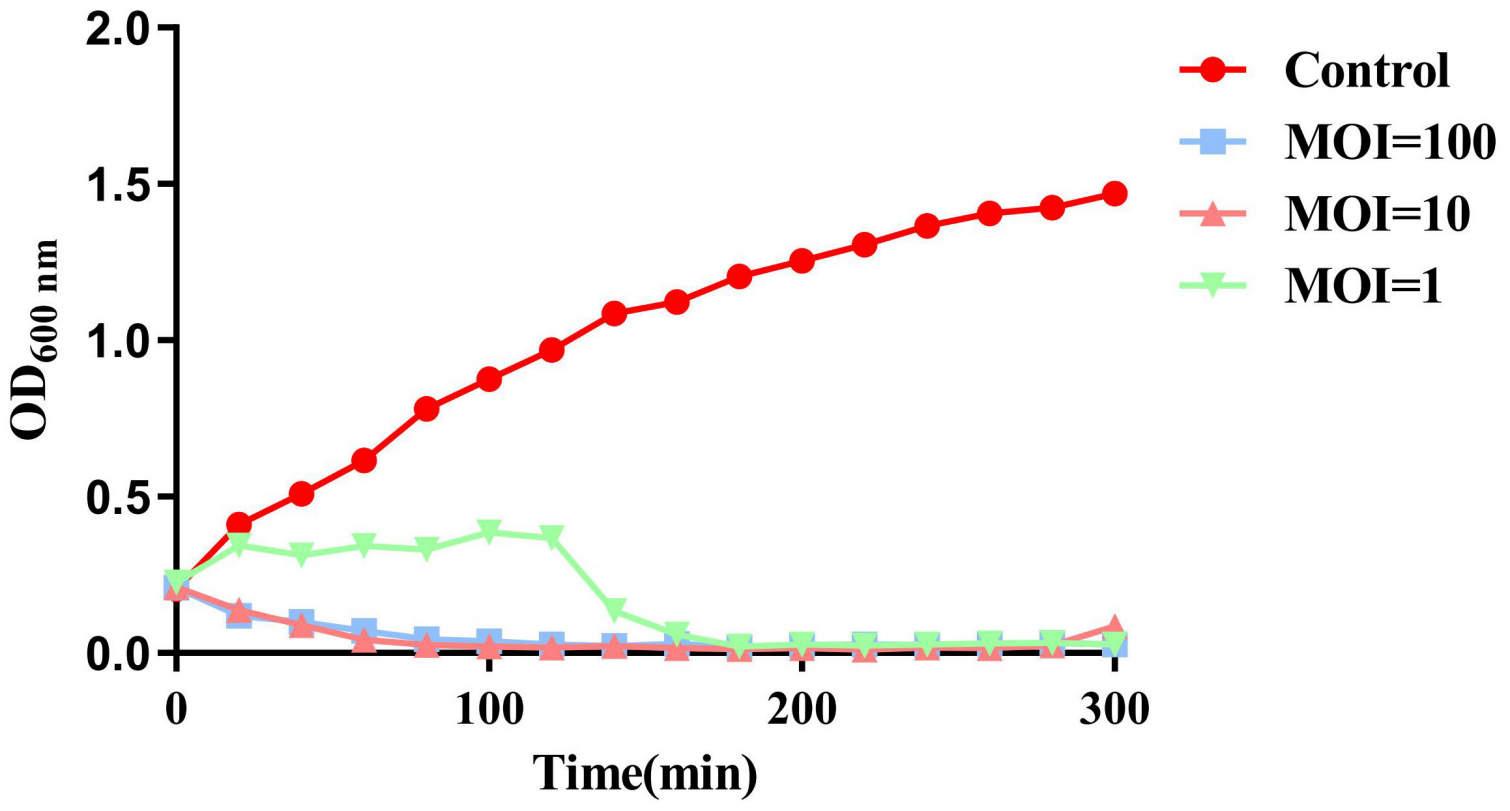
Antibacterial activity of phage SPB with different MOIs against MRSA.

To evaluate the inhibitory effect of phage SPB on planktonic bacteria, MRSA (B2-2) was cultured to an OD_600nm_ value of 0.2–0.3, and phage SPB was added at MOI of 100, 10, and 1. As illustrated in Figure 6, phage SPB was effective in inhibiting the growth of MRSA within 20 min at both MOI = 100 and MOI = 10, and had an inhibitory effect at both at 300 min, whereas phage SPB was effective in inhibiting the growth of MRSA only after 120 min of co-cultivation at MOI = 1.

### 3.8 Biofilm sensitivity to phage SPB

**Figure 7:**
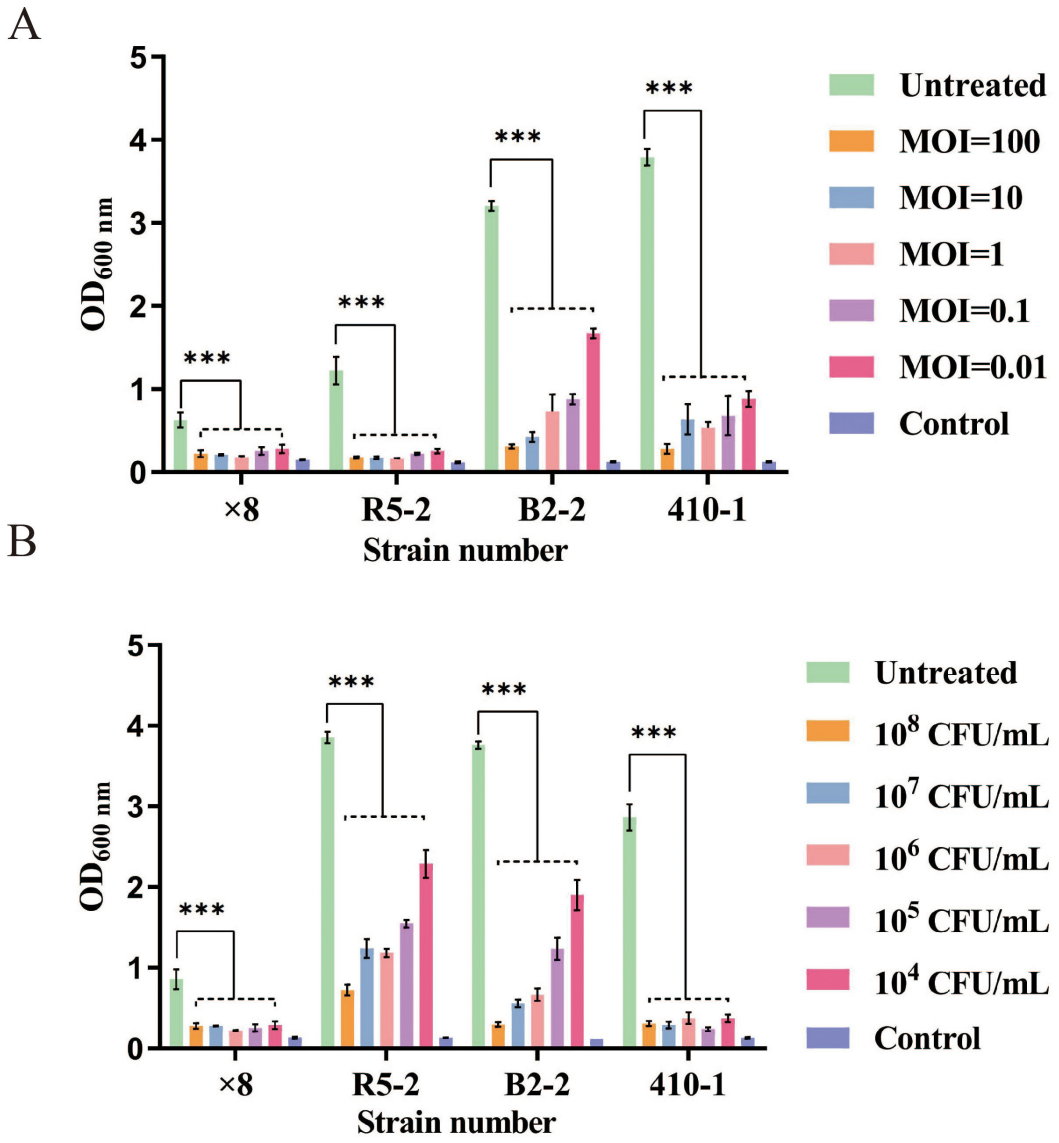
Inhibition and clearance of four strains of MRSA biofilm by phage SPB. ***P < 0.001. **(A)** Inhibition of biofilm formation by phage SPB. **(B)** Clearance of mature biofilm by phage SPB. ***P < 0.001.

To evaluate the ability of phage SPB to inhibit biofilm formation and eradicate mature biofilms formed by four MRSA strains. In the experiment aimed at inhibiting biofilm formation (Figure 7A), the phage SPB treatment group (MOI 100–0.01) demonstrated a significant reduction in the formation of biofilm in the four MRSA strains (*P* < 0.001) in a concentration-dependent manner. Compared to the control group, phage SPB decreased the biofilm formation rates of strains × 8, R5-2, B2-2, and 410-1 to as low as 28.62%, 13.10%, 9.67%, and 7.43%, respectively, resulting in biofilm inhibition rates of up to 71.38%, 86.90%, 90.33%, and 92.57%. In the biofilm scavenging assay (Figure 7B), phage SPB exhibited a significant scavenging effect on mature biofilms of all four MRSA strains at varying concentrations (10^6^-10^8^) (*P* < 0.001). When compared to the control group, the biofilm of MRSA strains × 8, R5-2, B2-2, and 410-1 were reduced by ~74.43%, 81.24%, 92.14%, and 91.70% in the phage SPB-treated group, respectively.

### 3.9 Removal of biofilm by phage at different concentrations as observed by fluorescence microscopy

**Figure 8:**
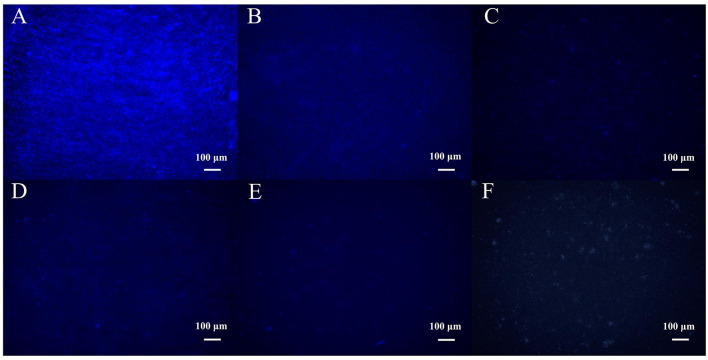
Fluorescence micrographs of biofilms formed after 18 h of treatment with different concentrations of phage SPB. **(A)** Mature biofilm formed in the absence of phage (e.g. SM buffered control group). **(B)** 10^4^ CFU/mL treatment group. **(C)** 10^5^ CFU/mL treatment group. **(D)** 10^6^ CFU/mL treatment group. **(E)** 10^7^ CFU/mL treatment group. **(F)** 10^8^ CFU/mL treatment group.

Fluorescence microscopy observation showed that MRSA strain 410-1 formed a dense biofilm structure (Figure 8A). The density of bacteria in the biofilm was significantly reduced after 18 h of treatment with different concentrations of phage SPB (Figures 8B–F). When the phage concentration was 10^8^ CFU/mL, the integrity of the bacterial biofilm structure was disrupted (Figure 8F). It indicates that phage SPB has a strong ability to kill bacteria and destroy the biofilm structure.

## 4 Discussion

*S.aureus* is increasingly developing resistance to many commonly used antibiotics, and is capable of forming biofilms, which makes the treatment of *S. aureus* increasingly difficult. Bacteriophages offer advantages in controlling multi-drug resistant bacteria and their associated biofilms (Jiang et al., 2021). However, most phages exhibit host specificity and possess a limited cleavage range, which constrains their application (Lu et al., 2023). Consequently, there is an urgent need to identify effective phages with a broad host range.

In this study, we identified a broad-spectrum MRSA phage capable of infecting 97.3% (36/37) of MRSA strains. In addition, phage SPB lysed *S. aureus* from 6 different animal sources (93.94%, 31/33) and 14 SPA types (90.00%, 54/60), as well as 10 strains of coagulase-negative *staphylococci*. The lysis range of phage SPB was broader compared to that of phage vB_SauP_ASUmrsa123 (El-Tawab et al., 2024). Phage SPB has an icosahedral head and retractable tail, similar to phage RuSa1 (Suchithra et al., 2025). According to previous reports, phage VL10 (Lerdsittikul et al., 2024) and SaGU1 (Shimamori et al., 2021) latencies were 35 min and 40 min. In contrast, phage SPB had a shorter latency, but the outbreak volume of phage SPB was lower. The phage remains relatively stable across a temperature range of 4°C to 50°C and pH levels between 4 and 11, suggesting that phage SPB is resilient to changes in environmental conditions during use. Additionally, phage SPB maintains stability at 4°C for over 2 months, facilitating convenient storage. Increasingly, phages are being used by the food industry as antibacterial agents or preservatives to control the growth of foodborne pathogens. Previous studies have demonstrated the successful application of bacteriophage products in agricultural, food safety, and diagnostic settings (Song et al., 2021).

The host cell lysis of most dsDNA phages upon completion of the replication cycle is primarily mediated by holin and lyase (Cahill and Young, 2019). Holin forms pores in the host cell's plasma membrane, allowing lyase access to the peptidoglycan layer, which it subsequently degrades, leading to rapid bacterial lysis (Fernandes and Sao-Jose, 2016). The holin-lyase system, a key mechanism in phage-induced lysis, is encoded by genes that are typically located adjacent to one another in the phage genome (Cahill and Young, 2019). Phage SPB, as identified in this study, contains genes encoding holin-lyase system and shares structural similarities with phage qdsa002 (Ning et al., 2021). The CRISPR-Cas system is an adaptive immune defense found in many bacteria and archaea. This system, composed of CRISPR arrays and associated Cas proteins, functions to recognize and eliminate foreign phage DNA, posing a challenge for phage therapy (Yosef et al., 2012). However, a novel study by Bondy-Denomy et al. (2013) described an anti-CRISPR-Cas protein complex in the Pseudomonas aeruginosa UCBPP-PA14 bacteriophage, which effectively prevents the host's CRISPR-Cas system from degrading phage DNA. In this study, three anti-CRISPR-Cas proteins were identified through PhageScope prediction. It is speculated that the phage SPB isolated in this research may have the capability to inhibit the host CRISPR-Cas immune system. However, this function requires further verification.

Phage products have been used in agriculture, food safety and diagnostic applications, confirming the utility and feasibility of phage applications (Song et al., 2021). In this study, in order to test the application potential of phage SPB, the inhibitory ability of phage on MRSA strains grown in a suitable environment was tested. The results showed that all experimental groups were effective in inhibiting the growth of MRSA strains within 300 min, which is consistent with the effect of phage isolated by Liu et al. (2022). All of them were able to effectively inhibit the growth of host bacteria within 300 min. However, it has been shown that phage-resistant strains can emerge when phages are used alone. To address this situation, mixing multiple phages significantly reduces the probability of phage-resistant strains appearing (Chen et al., 2024). Biofilms are considered a major problem for the medical and food industries, and also pose a threat to the environment (Vergara et al., 2017). *S. aureus* strains that form biofilms are generally more difficult to kill and more resistant to antimicrobials than bacterial strains that float (Lister and Horswill, 2014). Previous studies have shown that phages can significantly reduce bacterial populations and remove biofilms (Song et al., 2021). In this study, phage SPB could significantly inhibit biofilm formation and clear biofilm (*P* < 0.001). Fluorescence microscopy showed that the integrity of the biofilm structure was destroyed after phage SPB treatment. Therefore, bacteriophage SPB is expected to be an effective disinfectant or bactericide to remove MRSA biofilm in food processing.

## 5 Conclusion

MRSA poses a serious threat to food safety and is a major public health concern worldwide. Therefore, there is an urgent need to develop safe and reliable biological control methods to improve public health and ensure consumer safety. In this study, the virgous bacteriophage SPB isolated in the range of 4°C to 50°C and pH range of 4–11 can maintain relatively stable, short incubation period, wide cleavage range, and can significantly inhibit biofilm formation and significantly clear biofilm. Therefore, bacteriophage SPB is expected to be used to control MRSA contamination in food and environment.

## Data Availability

The datasets presented in this study can be found in online repositories. The names of the repository/repositories and accession number(s) can be found in the article/Supplementary material.

## References

[B1] Abolghait S. K. Fathi A. G. Youssef F. M. Algammal A. M. (2020). Methicillin-resistant Staphylococcus aureus (MRSA) isolated from chicken meat and giblets often produces staphylococcal enterotoxin B (SEB) in non-refrigerated raw chicken livers. Int. J. Food Microbiol. 328:108669. 10.1016/j.ijfoodmicro.2020.108669 32497922

[B2] Bondy-Denomy J. Pawluk A. Maxwell K. L. Davidson A. R. (2013). Bacteriophage genes that inactivate the CRISPR/Cas bacterial immune system. Nature 493, 429–432. 10.1038/nature11723 23242138 PMC4931913

[B3] Cahill J. Young R. (2019). Phage lysis: multiple genes for multiple barriers. Adv. Virus Res. 103, 33–70. 10.1016/bs.aivir.2018.09.003 30635077 PMC6733033

[B4] Chen H. Liu H. Gong Y. Dunstan R. A. Ma Z. Zhou C.. (2024). A Klebsiella-phage cocktail to broaden the host range and delay bacteriophage resistance both *in vitro* and *in vivo*. NPJ Biofilms Microbiomes 10:127. 10.1038/s41522-024-00603-8 39543151 PMC11564825

[B5] Cui Z. Feng T. Gu F. Li Q. Dong K. Zhang Y.. (2017). Characterization and complete genome of the virulent Myoviridae phage JD007 active against a variety of Staphylococcus aureus isolates from different hospitals in Shanghai, China. Virol. J. 14:26. 10.1186/s12985-017-0701-0 28179010 PMC5299689

[B6] Cuny C. Nathaus R. Layer F. Strommenger B. Altmann D. Witte W. (2009). Nasal colonization of humans with methicillin-resistant Staphylococcus aureus (MRSA) CC398 with and without exposure to pigs. PLoS One 4:e6800. 10.1371/journal.pone.0006800 19710922 PMC2728842

[B7] Devriese L. A. Van Damme L. R. Fameree L. (1972). Methicillin (cloxacillin)-resistant Staphylococcus aureus strains isolated from bovine mastitis cases. Zentralbl Veterinarmed. B. 19, 598–605. 10.1111/j.1439-0450.1972.tb00439.x 4486473

[B8] El-Tawab A. K. A. Othman B. A. Sharaf A. El-Masry S. S. El-Arabi T. F. (2024). Characterization and complete genome sequence of highly lytic phage active against methicillin-resistant Staphylococcus aureus (MRSA) isolated from Egypt. Virol. J. 21:284. 10.1186/s12985-024-02554-0 39516905 PMC11545979

[B9] Fernandes S. Sao-Jose C. (2016). More than a hole: the holin lethal function may be required to fully sensitize bacteria to the lytic action of canonical endolysins. Mol. Microbiol. 102, 92–106. 10.1111/mmi.13448 27328857

[B10] Graveland H. Duim B. van Duijkeren E. Heederik D. Wagenaar J. A. (2011). Livestock-associated methicillin-resistant Staphylococcus aureus in animals and humans. Int. J. Med. Microbiol. 301, 630–634. 10.1016/j.ijmm.2011.09.004 21983338

[B11] Gutierrez D. Rodriguez-Rubio L. Martinez B. Rodriguez A. Garcia P. (2016). Bacteriophages as weapons against bacterial biofilms in the food industry. Front. Microbiol. 7:825. 10.3389/fmicb.2016.00825 27375566 PMC4897796

[B12] Gutierrez D. Vandenheuvel D. Martinez B. Rodriguez A. Lavigne R. Garcia P. (2015). Two Phages, phiIPLA-RODI and phiIPLA-C1C, Lyse Mono- and dual-species Staphylococcal biofilms. Appl. Environ. Microbiol. 81, 3336–3348. 10.1128/AEM.03560-14 25746992 PMC4407228

[B13] Idelevich E. A. Lanckohr C. Horn D. Wieler L. H. Becker K. Kock R. (2016). [Multidrug-resistant bacteria in Germany. The impact of sources outside healthcare facilities]. Bundesgesundheitsblatt Gesundheitsforschung Gesundheitsschutz 59, 113–123. 10.1007/s00103-015-2261-z 26446586

[B14] Jauro S. Hamman M. M. Malgwi K. D. Musa J. A. Ngoshe Y. B. Gulani I. A.. (2022). Antimicrobial resistance pattern of methicillin-resistant Staphylococcus aureus isolated from sheep and humans in Veterinary Hospital Maiduguri, Nigeria. Vet. World 15, 1141–1148. 10.14202/vetworld.2022.1141-1148 35698509 PMC9178588

[B15] Jiang Y. Xu Q. Jiang L. Zheng R. (2021). Isolation and characterization of a Lytic Staphylococcus aureus phage WV against Staphylococcus aureus biofilm. Intervirology 64, 169–177. 10.1159/000515282 34229320 PMC8619764

[B16] Kabwe M. Brown T. Speirs L. Ku H. Leach M. Chan H. T.. (2020). Novel bacteriophages capable of disrupting biofilms from clinical strains of Aeromonas hydrophila. Front. Microbiol. 11:194. 10.3389/fmicb.2020.00194 32117183 PMC7033617

[B17] Kifelew L. G. Warner M. S. Morales S. Vaughan L. Woodman R. Fitridge R.. (2020). Efficacy of phage cocktail AB-SA01 therapy in diabetic mouse wound infections caused by multidrug-resistant Staphylococcus aureus. BMC Microbiol. 20:204. 10.1186/s12866-020-01891-8 32646376 PMC7346408

[B18] Kong X. Wang H. Guo G. Li P. Tong P. Liu M.. (2022). Duck sewage source coliphage P762 can lyse STEC and APEC. Virus Genes 58, 436–447. 10.1007/s11262-022-01915-7 35705841

[B19] Krzywinski M. Schein J. Birol I. Connors J. Gascoyne R. Horsman D.. (2009). Circos: an information aesthetic for comparative genomics. Genome Res. 19, 1639–1645. 10.1101/gr.092759.109 19541911 PMC2752132

[B20] Lakhundi S. Zhang K. (2018). Methicillin-resistant Staphylococcus aureus: molecular characterization, evolution, and epidemiology. Clin. Microbiol. Rev. 31, e00020–18. 10.1128/CMR.00020-18 30209034 PMC6148192

[B21] Lerdsittikul V. Apiratwarrasakul S. Atithep T. Withatanung P. Indrawattana N. Pumirat P.. (2024). Isolation and characterisation of a novel bacteriophage promising antimicrobial agent against methicillin-resistant infections. Sci. Rep. 14:9251. 10.1038/s41598-024-59903-w 38649443 PMC11035597

[B22] Li M. Guo M. Chen L. Zhu C. Xiao Y. Li P.. (2020). Isolation and characterization of novel lytic Bacteriophages infecting epidemic carbapenem-resistant Klebsiella pneumoniae strains. Front. Microbiol. 11:1554. 10.3389/fmicb.2020.01554 32793133 PMC7385232

[B23] Lister J. L. Horswill A. R. (2014). Staphylococcus aureus biofilms: recent developments in biofilm dispersal. Front. Cell Infect. Microbiol. 4:178. 10.3389/fcimb.2014.00178 25566513 PMC4275032

[B24] Liu B. Zheng D. Jin Q. Chen L. Yang J. (2019). VFDB 2019: a comparative pathogenomic platform with an interactive web interface. Nucleic. Acids Res. 47, D687–D692. 10.1093/nar/gky1080 30395255 PMC6324032

[B25] Liu S. Hon K. Bouras G. S. Psaltis A. J. Shearwin K. Wormald P. J.. (2022). APTC-C-SA01: a novel Bacteriophage cocktail targeting Staphylococcus aureus and MRSA biofilms. Int. J. Mol. Sci. 23:6116. 10.3390/ijms23116116 35682794 PMC9181636

[B26] Lu Y. Lu Y. Li B. Liu J. Wang L. Zhang L.. (2023). StAP1 phage: an effective tool for treating methicillin-resistant Staphylococcus aureus infections. Front. Microbiol. 14:1267786. 10.3389/fmicb.2023.1267786 37840707 PMC10570516

[B27] McNair K. Bailey B. A. Edwards R. A. (2012). PHACTS, a computational approach to classifying the lifestyle of phages. Bioinformatics 28, 614–618. 10.1093/bioinformatics/bts014 22238260 PMC3289917

[B28] Nandhini P. Kumar P. Mickymaray S. Alothaim A. S. Somasundaram J. Rajan M. (2022). Recent developments in Methicillin-resistant Staphylococcus aureus (MRSA) treatment: a review. Antibiotics 11:606. 10.3390/antibiotics11050606 35625250 PMC9137690

[B29] Nemati M. Hermans K. Lipinska U. Denis O. Deplano A. Struelens M.. (2008). Antimicrobial resistance of old and recent Staphylococcus aureus isolates from poultry: first detection of livestock-associated methicillin-resistant strain ST398. Antimicrob. Agents Chemother. 52, 3817–3819. 10.1128/AAC.00613-08 18663024 PMC2565892

[B30] Ning H. Lin H. Wang J. He X. Lv X. Ju L. (2021). Characterizations of the endolysin Lys84 and its domains from phage qdsa002 with high activities against Staphylococcus aureus and its biofilms. Enzyme Microb. Technol. 148:109809. 10.1016/j.enzmictec.2021.109809 34116743

[B31] Petrovic Fabijan A. Lin R. C. Y. Ho J. Maddocks S. Ben Zakour N. L. Iredell J. R.. (2020). Safety of bacteriophage therapy in severe Staphylococcus aureus infection. Nat. Microbiol. 5, 465–472. 10.1038/s41564-019-0634-z 32066959

[B32] Sadiq A. Samad M. Saddam B. Asharat N. Ali S. Roohullah. (2020). Methicillin-resistant Staphylococcus aureus (MRSA) in slaughter houses and meat shops in capital territory of Pakistan during 2018–2019. Front. Microbiol. 11:577707. 10.3389/fmicb.2020.577707 33117321 PMC7550752

[B33] Saginur R. Stdenis M. Ferris W. Aaron S. D. Chan F. Lee C.. (2006). Multiple combination bactericidal testing of staphylococcal biofilms from implant-associated infections. Antimicrob. Agents Chemother. 50, 55–61. 10.1128/AAC.50.1.55-61.2006 16377667 PMC1346774

[B34] Shimamori Y. Pramono A. K. Kitao T. Suzuki T. Aizawa S. Kubori T.. (2021). Isolation and characterization of a novel phage SaGU1 that Infects clinical isolates from patients with Atopic Dermatitis. Curr. Microbiol. 78, 1267–1276. 10.1007/s00284-021-02395-y 33638001 PMC7997843

[B35] Shkoporov A. N. Clooney A. G. Sutton T. D. S. Ryan F. J. Daly K. M. Nolan J. A.. (2019). The human gut virome is highly diverse, stable, and individual specific. Cell Host Microbe. 26, 527–541.e5. 10.1016/j.chom.2019.09.009 31600503

[B36] Song J. Ruan H. Chen L. Jin Y. Zheng J. Wu R.. (2021). Potential of bacteriophages as disinfectants to control of Staphylococcus aureus biofilms. BMC Microbiol. 21:57. 10.1186/s12866-021-02117-1 33607940 PMC7896381

[B37] Stefani S. Chung D. R. Lindsay J. A. Friedrich A. W. Kearns A. M. Westh H.. (2012). Meticillin-resistant Staphylococcus aureus (MRSA): global epidemiology and harmonisation of typing methods. Int. J. Antimicrob. Agents 39, 273–282. 10.1016/j.ijantimicag.2011.09.030 22230333

[B38] Suchithra K. V. Hameed A. Rekha P. D. Stothard P. Arun A. B. (2025). A novel Kayvirus species phage RuSa1 removes biofilm and lyses multiple clinical strains of methicillin resistant Staphylococcus aureus. Sci. Rep. 15:7358. 10.1038/s41598-025-92032-6 40025202 PMC11873149

[B39] Turner N. A. Sharma-Kuinkel B. K. Maskarinec S. A. Eichenberger E. M. Shah P. P. Carugati M.. (2019). Methicillin-resistant Staphylococcus aureus: an overview of basic and clinical research. Nat. Rev. Microbiol. 17, 203–218. 10.1038/s41579-018-0147-4 30737488 PMC6939889

[B40] Vergara A. Normanno G. Di Ciccio P. Pedonese F. Nuvoloni R. Parisi A.. (2017). Biofilm Formation and its relationship with the molecular characteristics of food-related methicillin-resistant Staphylococcus aureus (MRSA). J. Food Sci. 82, 2364–2370. 10.1111/1750-3841.13846 28892140

[B41] Yang H. Xu J. Li W. Wang S. Li J. Yu J.. (2018). Staphylococcus aureus virulence attenuation and immune clearance mediated by a phage lysin-derived protein. EMBO J. 37:e98045. 10.15252/embj.201798045 30037823 PMC6120661

[B42] Yang M. Chen H. Huang Q. Xie Z. Liu Z. Zhang J.. (2022). Characterization of the novel phage vB_VpaP_FE11 and its potential role in controlling vibrio parahaemolyticus biofilms. Viruses 14:264. 10.3390/v14020264 35215857 PMC8879856

[B43] Yosef I. Goren M. G. Qimron U. (2012). Proteins and DNA elements essential for the CRISPR adaptation process in *Escherichia coli*. Nucleic. Acids Res. 40, 5569–5576. 10.1093/nar/gks216 22402487 PMC3384332

[B44] Zhang L. Shahin K. Soleimani-Delfan A. Ding H. Wang H. Sun L.. (2022). Phage JS02, a putative temperate phage, a novel biofilm-degrading agent for Staphylococcus aureus. Lett. Appl. Microbiol. 75, 643–654. 10.1111/lam.13663 35100443

[B45] Zhang Q. Xing S. Sun Q. Pei G. Cheng S. Liu Y.. (2017). Characterization and complete genome sequence analysis of a novel virulent Siphoviridae phage against Staphylococcus aureus isolated from bovine mastitis in Xinjiang, China. Virus Genes 53, 464–476. 10.1007/s11262-017-1445-z 28299517

